# Lexical Orthographic Knowledge Mediates the Relationship Between Character Reading and Reading Comprehension Among Learners With Chinese as a Second Language

**DOI:** 10.3389/fpsyg.2022.779905

**Published:** 2022-03-31

**Authors:** Xian Liao, Elizabeth Ka Yee Loh, Mingjia Cai

**Affiliations:** ^1^Department of Chinese Language Studies, The Education University of Hong Kong, Tai Po, Hong Kong SAR, China; ^2^Faculty of Education, University of Hong Kong, Pokfulam, Hong Kong SAR, China

**Keywords:** lexical orthographic knowledge, character reading, reading comprehension, Chinese as a second language, mediating effect

## Abstract

Reading in Chinese is complex because readers should not only recognize characters by basic units (i.e., characters) but also integrate characters into words when reading text. While many efforts have been devoted to investigating the effect of sub-lexical orthographic knowledge in Chinese character reading, less is known about the role played by lexical orthographic knowledge at word level. A total of 424 secondary learners with Chinese as a second language (CSL) in Hong Kong were assessed with character reading, reading comprehension, and two lexical orthographic knowledge tasks: lexical orthographic choice (OKC) and lexical orthographic choice in context (OKCC). Path analysis results demonstrated that these lexical orthographic skills significantly mediated the effect of character reading on comprehension, in which OKCC was a more critical mediator as its mediating effect was bigger than that of OKC. Further analyses showed that these mediating effects were significant only among students with richer learning experience (i.e., learning Chinese for 4 years or above). Our results illustrate the possible trajectory of CSL learners’ literacy development from character reading to reading comprehension and provide pedagogical implications for teaching and learning.

## Introduction

The growing number of learners with Chinese as a second language (CSL) (refer to CSL hereafter) worldwide in recent decades has been driving the research in exploring factors accounting for students’ individual difference in Chinese learning (Gong et al., 2018, 2020a,b). In Hong Kong, there were about 33,000 ethnic-minority students who are mostly descendants of migrants from India, Pakistan, Nepal, and the Philippines in 2019–2020 academic year, consisting 4.5% of the total school-aged children population (Legislative Council of Hong Kong SAR, 2020). These students attend local kindergartens and primary/secondary schools but mostly take Chinese as a second language. Consequently, their reading proficiency significantly lags behind their Chinese counterparts for about 3–4 years, which largely undermines their confidence in Chinese language learning and in turn hinders their academic achievements, social integration and upward social mobility (Leong et al., 2011; Loh and Tam, 2016). To address the pressing need, teachers and researchers have been seeking effective and tailor-made approaches derived from empirical evidence about CSL students’ characteristics in learning Chinese (Gong et al., 2020b).

The learning of Chinese usually starts from recognizing characters as it is the basic unit representing meaning and syllable in Chinese. Meanwhile, each Chinese character is also visually complex. Thus previous studies have suggested that mastery of Chinese orthographic rules could be important to character recognition (Loh et al., 2017; Wong, 2019; Chan et al., 2021) or reading comprehension among CSL learners (Shen, 2013; Wong, 2019; Dong et al., 2020). However, most of these studies have focused on character-level orthographic knowledge (i.e., sub-lexical orthographic knowledge), and surprisingly another type of orthographic knowledge, i.e., lexical orthographic knowledge, was ignored. In fact, character reading does not necessarily ensure a successful word reading because plenty of words in Chinese comprised of two or more characters (i.e., morphemes), and their meaning are usually holistically bound. The word reading process is found rather different from that of single characters reading in terms of the possible skills involved (Li and McBride-Chang, 2014; Pan et al., 2021). In this case, lexical orthographic knowledge was assumed to be employed by learners to integrate characters into words and achieve a smooth and fluent recognition of words during reading.

In this study, we hypothesized that lexical orthographic knowledge could mediate the relationship between character reading and reading comprehension among CSL learners. We also explored whether students’ length of experience in learning Chinese affects this mechanism. To our knowledge, this is the first study dedicated to the role of Chinese lexical orthographic knowledge in reading comprehension, which may not only contribute to the theoretical studies on the second language but also shed light on important practical implications for CSL learning and teaching.

## Literature Review

### Basic Properties of Chinese Writing System

With characters as the basic graphic unit, the Chinese writing system is a morpho-syllabic writing system that distinctively differs from alphabetic languages (Wang et al., 2003; Perfetti et al., 2005; Shen, 2005; Tong et al., 2009; Li et al., 2012; Lü and Koda, 2017). In other words, each character represents a morpheme that is a unit with specific syllable and meaning. Each Chinese character is a two-dimensional, visual-spatial unit (Cheung et al., 2006) and most Chinese characters (96%) are compound characters, consisting of at least two radicals (Su, 2001). The radical is a stroke pattern that hints full or partial cues of sound/meaning of a character (i.e., phonologically or semantically related to the morpheme represented by the character), though the indication accuracy is sometimes rather weak (Zhou, 1978). Additionally, some radicals are with fixed positions (Taylor and Taylor, 2014). For instance, the semantic radical 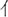 [jan4, human being] only appears on the left of a character.

The term of words in Chinese language is complicated (e.g., Packard, 2000). Although a number of single morphemes are words (such as 水 [*water*]), most Chinese words (around 80%) are polymorphemic, which means they contain two or more characters, making them a so-called *complex word* (Perfetti and Tan, 1999; Ke and Koda, 2019). Whereas some two-character words’ meaning could be inferred by combining the meaning of each character (e.g., the meaning of 升起 *[uprising]* can be retrieved from the two morphemes 升 *[up]* and 起 *[rise])*, the meaning of many polymorphemic word is somewhat opaque and may not always be equal to the sum of its component morphemes (Gong et al., 2020b). For example, the meaning of 一起 *[together]* cannot be obtained in the same way as 升起 (i.e.,一 *[one]* + 起*[rise]*).

Furthermore, boundaries between words in written Chinese are not as salient as in alphabetic languages. For instance, 桌上擺滿各種可口的食物 [*There are many delicious foods on the table*] contains several words, i.e., 桌上 *[on the table]*/擺滿 *[placing]*/各種 *[various]*/可口 *[delicious]*/的 *[aux.]*/食物 *[foods]*. Though arrayed in line, characters are with syntagmatic relations, rather than linearly related. Therefore, to process sentences, readers need to segment words in a string of characters, which requires word and word-constituent knowledge at both form and meaning levels (Li et al., 2009; Li and Pollatsek, 2020; Cui et al., 2021).

### Lexical Orthographic Knowledge in Chinese Reading

Orthographic knowledge is commonly considered to benefit word recognition and subsequent reading comprehension across languages (e.g., Burt, 2006; Leong et al., 2011, 2019; Wong, 2017; Deacon et al., 2019; Zarić et al., 2020). In alphabetic languages such as English, there are generally two separate but correlated types of orthographic knowledge, one is called “general/sub-lexical orthographic knowledge” (e.g., identifying the letter patterns that violate the orthographic regularities, such as “bbaf”), and the other is called “lexical orthographic knowledge” (e.g., choosing the correct word form between “rain” and “rane”) (Apel, 2011; Conrad et al., 2013; Rothe et al., 2015).

In response to the complexity of Chinese orthography, sub-lexical orthographic knowledge could be defined as one’s knowledge of legal character forms, including positional knowledge and functional knowledge of radicals (Ho et al., 2003; Loh et al., 2018; Chan et al., 2021). Taking the character “花 [faa1, flower]” as an example, a learner with rich sub-lexical orthographic knowledge not only notices that the radical “艹” [plant] only appears at the top of a character, but also understand that a character with “艹” may be plant-related in meaning. For CSL learners, it has been well documented that they rely more on sub-lexical orthographic knowledge to recognize characters than their Chinese native counterparts do (Shen and Ke, 2007; Chang et al., 2014; Wong, 2017; Loh et al., 2018; Chan et al., 2021).

As another important type of Chinese orthographic knowledge, the lexical orthographic knowledge is defined as the knowledge of writing conventions of complex words, including the written form of word constituents and their combinations (Leong et al., 2011, 2019; Conrad et al., 2013). Given the fact that it has been less studied, we referred to the dual-route model (Coltheart et al., 2001) to explore the role of lexical orthographic knowledge. According to this model, words could be recognized from two routes, namely the sub-lexical route and the lexical route. For the sub-lexical route, readers retrieve the meaning/sound of a word analytically to integrate sub-lexical information. Therefore, the sub-lexical orthographic knowledge is assumed to support learners to decode Chinese words in this way. For instance, readers employ such knowledge to recognize morphemes 花 [faa1, flower] and 瓶 [peng4, bottle] individually and linearly, so that they form the word 花瓶 [faa1 peng4, lit. vase] and get the meaning of the word.

On the contrary, lexical orthographic knowledge would be necessary for decoding words from a direct and lexical route. Researchers tended to argue that with this knowledge, readers could automatically process the familiar word-form as a single unit and access the meaning/sound of the word directly, so that they could quickly and fluently comprehend text at higher linguistic levels (e.g., sentence and text levels) (Conrad et al., 2013; Zarić et al., 2020). In considering the fact that there are a large number of Chinese words whose meanings are more or less opaque, the analytical approach may not be applicable in reading these words, therefore, the lexical orthographic knowledge could help readers to quickly recall the correct meaning of each word in reading texts. Recent studies conducted among primary students (Chu and Leung, 2005) and undergraduate students (Cui et al., 2021) have also suggested that the Chinese native students tended to adopt a holistically processing in reading frequent words. In a similar way, we could assume that with more knowledge in Chinese gained in learning, the CSL learners could be more familiar with the holistically processing.

Furthermore, the lexical orthographic knowledge is assumed to help CSL learners to recognize the frequent words and access the meaning quickly when reading larger linguistic units (e.g., sentence or above). Because the boundary between words is inconspicuous, the more lexical orthographic knowledge gained, the better CSL learners could holistically read the words and understand the texts. To our knowledge, only Leong et al. (2011) used two tasks to measure a group of CSL (*N* = 80) students’ lexical orthographic knowledge, i.e., *orthographic choice task* and *orthographic choice in context*, corresponding to lexical orthographic knowledge at word- and sentence-level, respectively. They found that students’ performance in *orthographic choice in context* could significantly predict their reading comprehension performance. However, the complicated role of lexical orthographic knowledge during reading comprehension was not investigated.

### The Mediating Role of Lexical Orthographic Knowledge in the Relationship Between Chinese Character Reading and Reading Comprehension

As the character is the basic unit of Chinese writing system, character reading is a fundamental skill to decode Chinese texts. The significant association between character reading and reading comprehension in Chinese has been well documented although the character reading tasks used in the literature were not consistent (Joshi et al., 2012; Shen and Jiang, 2013; Yeung et al., 2013a; Wong, 2017; Yan et al., 2020). For instance, Yeung et al. (2013a) pointed out that word reading (i.e., two-character words) accounted for 33% of the variance in total reading comprehension scores among 248 Chinese Primary 4 students in Hong Kong after controlling for age and IQ. Shen and Jiang (2013) found a moderate-high positive correlation between students’ performance on the reading comprehension test and single character-reading accuracy (*r* = 0.64) among a small group of first-year CSL adult learners (*N* = 44). Besides, Wong (2017) conducted a longitudinal study among a sample of 142 Grade 4 CSL students in Hong Kong and found that students’ character reading (i.e., the total score of both one-character and two-character reading) significantly correlated with their reading comprehension concurrently and in the long run (*r* = 0.78–0.81 in Grade 4 and Grade 5, respectively).

As noted, the single-character and two-character reading were interchangeably used in previous studies. With increasing evidence suggesting that the skills required for them could be different (e.g., Wang and McBride, 2016; Lo et al., 2019; Pan et al., 2021), the exact effect of character reading on comprehension among a larger group of CSL learners should be examined. Furthermore, in-depth exploration should also go beyond the simple correlation or regression results to reveal the mechanism of how character reading contributes to reading comprehension.

In recognizing the importance of lexical orthographic knowledge in word reading, we hypothesized that such knowledge could mediate the effect of character reading on comprehension among CSL learners. Since the characters only represent morphemes in Chinese thus the character reading is not sufficient to decode words, lexical orthographic knowledge could thus assist readers to recognize specific word forms in a string of characters. To our understanding, first, the character reading skill is expected to predict the lexical orthographic knowledge since it provides a foundation for readers to recognize the constituents of words; second, this knowledge is anticipated to facilitate readers to access the meaning of a word more speedily through the familiar word form based on holistic word recognition, contributing to a fluent literal understanding for comprehension. If these associations are significant, we could expect a significant mediating effect played by the lexical orthographic knowledge.

### Individual Difference in Lexical Orthographic Knowledge’s Mediating Effect Among Students With Different Lengths of Learning Experience

Theoretically, the length of learning experience has been well acknowledged as a critical predictor of L2 proficiency (Sasaki, 1991). However, to what extent that the learning experience impacts the CSL learning needs a more comprehensive investigation (Conti and Lepadat, 2021). The learning experiences of Hong Kong CSL learners are very diverse. Many of them came to Hong Kong after partial or full completion of primary school in their respective countries. Due to administrative arrangements, they are usually allocated to the same class with peers who have completed primary school in Hong Kong with a fluent command of conversational Chinese, regardless of their limited Chinese learning experiences. As a result, students’ length of learning experience could be significantly different even though they are the same age and learn Chinese in a same classroom. This provided us with a unique opportunity to examine whether the individual differences in the length of learning experience could affect the relationships between orthographic skills and language skills, such as character reading and reading comprehension.

In the present study, we assumed that the direct and indirect effect of character reading on comprehension could be varied among students with different lengths of learning experience. The hypothesis was grounded on multiple pieces of evidence found in Chinese L1 students. First, the effect of character reading on comprehension may not be static, although the results were not conclusive. For instance, Joshi et al. (2012) found that the explained variance of reading comprehension by character recognition measured by pinyin writing task increased from Grade 2 (22%) to 4 (32%). On the contrary, Yan et al. (2020) found that the contribution of single character reading to reading comprehension decreased from Grade 1 (29%) to Grade 3 (8%). Second, the relationship between the orthographic knowledge on comprehension could also be varied. Yeung et al. (2013b) showed that orthographic knowledge of children in Grade 1 did not have a strong direct effect on character/word reading, whereas, in Grade 2–4, the effect became significant. Third, the acquisition of lexical orthographic knowledge could be partially contributed to the increase of learning experience. Generally, it is believed that with increasing experience of language learning (e.g., reading experience), both quality and quantity of lexical representations improve (Perfetti, 1992), which would allow learners to form better lexical orthographic knowledge. For instance, lower form students in Hong Kong tended to take the character level strategies to read two-character words while higher form students (Grade 5) were able to read these words holistically (Chu and Leung, 2005). While these grade differences are similar to the length of learning experience in CSL learners, one would anticipate that as students have accumulated more lexical orthographic knowledge, the mediating effect of lexical orthographic knowledge in students with more learning experience could therefore be stronger.

## The Present Study

Based on the literature outlined above, we tried to address the following two research questions: (1) Does CSL students’ Chinese lexical orthographic knowledge mediate the relationship between character reading and comprehension? If yes, (2) do the mediating effects of lexical orthographic knowledge vary between groups of CSL learners with different lengths of learning experiences?

To measure students’ lexical orthographic knowledge, we followed Leong et al. (2011) and adapted two lexical orthographic knowledge tasks used in their study. The orthographic choice task (OKC) and orthographic choice in context (OKCC) tasks were used to capture CSL students’ lexical orthographic knowledge at word- and sentence-levels respectively. In constructing the path model demonstrating the direct and indirect effects of character reading on comprehension, we put the OKC as the first mediator followed by the OKCC as the second one based on the following assumptions: (a) Following a bottom-up approach in reading (Gough, 1972; LaBerge and Samuels, 1974), reading is a process starting from the recognition of the smallest linguistic units (i.e., character) to larger units (words, clauses, sentences, and paragraphs). As hypothesized in the literature, the lexical orthographic knowledge could help readers surpass character-level reading and achieve quickly and fluently comprehend text at higher linguistic levels such as sentence- and text-levels (Ziegler et al., 2003; Jiang et al., 2020). Therefore, it was reasonable to arrange the variables with regard to the fine-grained linguistic units; (b) In terms of task difficulty, we considered OKCC was more challenging than OKC to CSL students because they had to additionally recognize characters and understand the context of sentences in order to complete the task.

### Participants

The present study was conducted among a group of CSL learners in Hong Kong. A total of 424 Secondary 1 CSL students (216 males and 208 females) from six secondary schools which admitted a large number of ethnic minority (EM) students were voluntarily recruited. According to the data of a self-reported language background questionnaire, students’ age ranged from 11 to 18 years old, *M* = 13.73, *SD* = 1.41. Among them, there were Filipinos (*n* = 50), Pakistanis (*n* = 184), Nepalese (*n* = 115), Indian (*n* = 45), and others (e.g., Brazilian, Bengalis, Nigerian, Russian, Thai, *n* = 30). All reported being mentally normal without dyslexia problems.

The mean of “Years learned Chinese” reported by the students is 7.23 (*SD* = 3.13). In considering the varied length of learning experiences, we decided to classify the participants into two groups by using mean minus an SD, which allowed us to purposely select the extremes of the distribution in the length of experience (i.e., the students with least learning experience) so that we could further determine the possible minimum requirement in the length of learning for the mediating effect. This classification method has also been adopted to identify the lower achievers in other reading-related studies (e.g., MacArthur et al., 2012; Duff and Brydon, 2020). As a result, the low experience group was for students with 4 years (or less) of experience in learning Chinese (*n* = 81), and the other was for those with more than 4 years of learning experience (*n* = 343).

### Measures

#### Sub-Lexical Orthographic Knowledge

This task was adapted from Wang et al. (2003) that was used to measure students’ sub-lexical orthographic knowledge. Students were required to determine whether the character shown on a card was a real character or not. The items were manipulated in four types of characters: (1) high frequency single characters, (2) high frequency compound characters; (3) low-frequency single characters; (4) low-frequency compound characters. 40 characters were selected for each category with a total of 160 items. Characters were selected from *Frequency statistics of commonly used Modern Chinese characters* (Ho and Kwan, 2001). 50% of these items were real characters, and the remaining 50% were non-characters. The frequency effect and number of radicals were matched for the four aforementioned types of characters. It took the students about 15 mins to complete the task. One point was given to each correct item. The Cronbach’s alpha was 0.955.

#### Lexical Orthographic Knowledge

There were two tasks used to measure students’ lexical orthographic knowledge: *orthographic choice task* (OKC) and *orthographic choice in context (OKCC).*

(1) The OKC task was used to measure students’ lexical orthographic knowledge without context. The original task was from Olson et al. (1985), consisting of one real English word and one homophonic pseudoword with a similar word shape (e.g., soap-sope; gawn-gone). Leong et al. (2011) modified it into the Chinese version. Adapted from Leong et al. (2011), we set 20 pairs of two-character words. These 20 questions consisted of (a) 10 pairs of words for each with one simple character and one regular consistent character (i.e., characters that are pronounced in the same way as its phonetic radical in tone and syllable, like 蜻 [cing1, dragonfly]), e.g., 青山 [cing1 saan1, green mountain] – 蜻山 [cing1 saan1, homophonic pseudoword]; (b) 5 pairs of words for each with one simple character and one regular inconsistent character (i.e., characters that are pronounced the same way as the correct answer; they shared the same phonetic radical but with different tones, like 飯 [faan6, meal]), e.g., 米飯 [mai5 faan6, rice] – 米反 [mai5 faan2, homophonic pseudoword]; and (c) 5 pairs with irregular or exception characters (i.e., characters that are pronounced in completely different ways from its phonetic radical, such as 直線 [zik6 sin3, line] – 直練 [zik6 lin6, homophonic pseudoword]. All characters and words were randomly selected from the Lexical Items for Fundamental Chinese Learning in Hong Kong Schools (Hong Kong Education Bureau, 2003)^1^. Each item contained two pairs of words, one being a real word, and the other a pseudoword. Students were required to read 20 pairs of word silently that were printed on a sheet of paper and circle the correct options. The task took about 10 mins to complete, and the maximum score was 20. The Cronbach’s alpha was 0.683.

(2) The *OKCC* task was used to measure the students’ lexical orthographic knowledge in context. It was first designed by Stanovich and West (1989) and the Chinese version was developed by Leong et al. (2011). This task consisted of 18 short sentences in total, with a maximum score of 18. Students were asked to read each Chinese short sentence silently and circle the only correct word among four options to complete the sentence meaning. Options consisted of two-character words, with three distractors that were orthographically or phonologically similar words of regular consistent, regular inconsistent, exception real, or pseudowords. For example: 早上(洋光/陽光/揚光/羊光) 照進課室 [ocean-light (pseudoword)/sunshine/blowing-light (pseudoword)/ sheep-light (pseudoword) shines into the classroom in the morning]. All characters and words were randomly selected from the Lexical Items for Fundamental Chinese Learning in Hong Kong Schools (Hong Kong Education Bureau, 2003; see text footnote 1). The testing time was 15 mins. One point was given to each correct item. The Cronbach’s alpha was 0.678.

#### Chinese Character Reading

The test consisted of 86 traditional single characters. All characters were randomly selected from textbooks used in the sampled schools, considering first the character frequency and then the complexity of strokes constituted the characters. The test was designed as an individual test, and participants were required to read aloud all characters one by one, following the instructions given by a trained test administrator. Each correct pronunciation was awarded one mark. The Cronbach’s alpha of the task was 0.980. The test items are listed in Appendix.

#### Chinese Reading Comprehension

Students’ reading comprehension ability was measured by using Chinese Language Reading Papers of the Territory-wide System Assessment (TSA). TSA is a low stake assessment measuring students’ basic literacy at the end of each key learning stage (i.e., Grade 3, 6, and 9, respectively) administered to all students in Hong Kong every year. For CSL students, we ascertained that their levels of reading and writing in Chinese were at about Grade 3 level based on literature (Loh et al., 2019) and teachers’ observation. Therefore, the TSA paper for Grade 3 students was adopted.

Students were required to read two passages (465 and 487 Chinese characters respectively) and answer 20 questions with 20 marks (one mark awarded for each correct answer) in 25 mins. The paper and the marking scheme were provided by the Hong Kong Examinations and Assessment Authority (2019), only single- or multiple-choice questions were included. The following three levels of reading competence were examined (Basaraba et al., 2013): (1) literal comprehension (retrieving explicitly stated information), eight questions; (2) inferential comprehension (understanding the implicit relationships in the passage), nine questions; and (3) evaluative comprehension (analyzing and critically interpreting the text), three questions. All scores were validated by two experienced Chinese language teachers. The Cronbach’s alpha for the test was 0.697.

### Procedure and Data Analysis

All scripts were marked by two raters with rich knowledge in Cantonese phonology who were trained prior to the tests. A sample of 40 scripts of each task were marked for trials, and discrepancies were resolved before formal marking. Since most of the items in all tasks were objective items (e.g., multiple-choice items), the inter-rater reliability as measured by Spearman rank-order correlation coefficients (rho) were relatively high, *r* ranging from 0.920 to 0.960.

All data were input into SPSS 26. Preliminary data analysis was first performed to check data adequacy, such as normality and outliers. The *t*-test was performed to examine the differences between both groups in all variables of this study. Due to the unequal sample sizes of the two learning experience groups, we calculated the Cohen’s *d* effect size. Cohen’s *d* between 0.2 and 0.4 represented small effects; between 0.5 and 0.7 medium effects; and above 0.8 large effects.

Path analysis was performed to explore the possible mediating effect of two lexical orthographic skills. It allowed us to simultaneously examine the structural relationships between variables and determine the direct and indirect paths. The indirect effect and mediating effect could be used interchangeably under different circumstances in this study. The maximum likelihood method was selected as it generated the estimation of all model path coefficients and to compute model fit statistics. To determine the significance of the indirect effects in each model, the bias-corrected bootstrapping was performed by 2,000 random sampling with replacements at a confidence level of 95%. The indirect effects were assumed significant when zero was beyond the confidence interval.

After the path model was built, a further multiple-group comparison was conducted to test whether the model structure was invariant for both groups. Following the procedure recommended by Byrne (2013), we compared a series of models to determine the paths that were variant between groups.

## Results

### Descriptive Analysis

Students’ performance in five tasks is presented in Table 1. Prior to further analysis, we checked the values of skewness and kurtosis and found that data of all variables was considered normally distributed, as the absolute values of skewness were less than 2 and kurtosis were below 5 (Kline, 2015).

**TABLE 1:** Descriptive data of students’ performance in four tasks.

Item (Max. scores)	All sample (*n* = 424)		Low experience (*n* = 81)		High experience (*n* = 341)		*t*-test	Cohen’s *d*
			
	Mean	*SD*	Mean	*SD*	Mean	*SD*		
1 Sub-lexical orthographic knowledge (160)	135.39	20.04	125.25	24.19	137.78	18.16	*t* = −4.38, *df* = 102.30, *p* < 0.001	0.64
2 OKC (20)	14.00	3.30	12.48	3.34	14.36	3.19	*t* = −4.73, *df* = 422, *p* < 0.001	0.58
3 OKCC (18)	6.81	3.24	5.58	2.47	7.10	3.33	*t* = −4.64, *df* = 156.03, *p* < 0.001	0.52
4 Character reading (86)	26.94	19.20	15.89	15.30	29.55	19.11	*t* = −6.87, *df* = 145.25, *p* < 0.001	0.74
5 Reading Comprehension (20)	4.66	3.10	3.82	2.20	4.86	3.25	*t* = −3.46, *df* = 173.37, *p* = 0.001	0.34

*Digits in the brackets are total number of tasks. OKC, lexical orthographic choice; OKCC, lexical orthographic choice in context.*

Students’ performance in all tasks was relatively poor. Students with more than 4 years’ learning experience outperformed their counterparts with less experience in all tasks, and all *p*-values of *t*-test were less than 0.05.

### Correlations Between Students’ Character Reading, Orthographic Knowledge and Reading Comprehension

Table 2 presents correlation coefficients among variables after controlling the age effect. As seen in Table 2, they were correlated with each other significantly at a moderate to high level. The reported length of learning experience in Chinese also correlated significantly with other tasks. However, the magnitude was at a weak to moderate level, r ranging from 0.103 to 0.247.

**TABLE 2:** Partial correlation between performances of four tasks after controlling age effect.

	1	2	3	4	5	6
1 Sub-lexical OK	1					
2 OKC	0.415***	1				
3 OKCC	0.303***	0.527***	1			
4 Character reading	0.405***	0.627***	0.614***	1		
5 Reading comprehension	0.206***	0.426***	0.508***	0.515***	1	
6 Years learned Chinese	0.209***	0.192***	0.164**	0.247***	0.103*	1

***p < 0.001, **p < 0.01, *p < 0.05.

*Sub-lexical OK, Sub-lexical orthographic knowledge; OKC, Orthographic choice; OKCC, Orthographic choice in context.*

### Direct and Indirect Effects of Character Reading on Comprehension

To explore the possible mediating effect of lexical orthographic knowledge in the relationship between character reading and reading comprehension, we first conducted a regression analysis using character reading to predict reading comprehension. The results showed that character reading significantly explained a total of 27.8% variance of reading comprehension, *F*(1, 422) = 162.38, *t* = 12.74, β = 0.527. This could be considered as the total effect of character reading.

**FIGURE 1:**
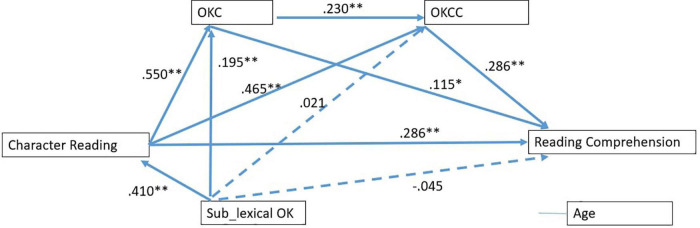
Path model demonstrating relationship between character reading and reading comprehension. **p< 0.01, *p< 0.05, Sub-lexical OK, Sub-lexical orthographic knowledge; OKC, Orthographic choice; OKCC, Orthographic choice in context. Coefficients for the controlled variable age were not shown for simplicity.

Next, we built a path model as hypothesized (see Figure 1) using two lexical orthographic knowledge tasks as mediators. Sub-lexical orthographic knowledge and age were added as controlled variables. By explaining a total of 34% variance of reading comprehension, the model was saturated. All standardized coefficients between variables were considered significant. As seen in Figure 1, character reading significantly predicted the two types of lexical orthographic knowledge, which further had positive effects on reading comprehension. Noted the direct effect of character reading on reading comprehension decreased from 0.527 to 0.286, which means the total indirect effect of lexical orthographic knowledge was about 0.241.

After decomposing the indirect effects into three pathways (see Table 3), we found that the indirect effect of character reading via OKC was significant, β = 0.063, *p* = 0.025, and OKCC significantly mediated the effect of character reading on reading comprehension (β = 0.133, *p* = 0.001) and could form a significant two-stage indirect effect (β = 0.036, *p* = 0.001) together with OKC, suggesting the more important role of OKCC.

### Difference in the Relationship Between Variables Among Two Groups With Different Learning Experiences

**FIGURE 2:**
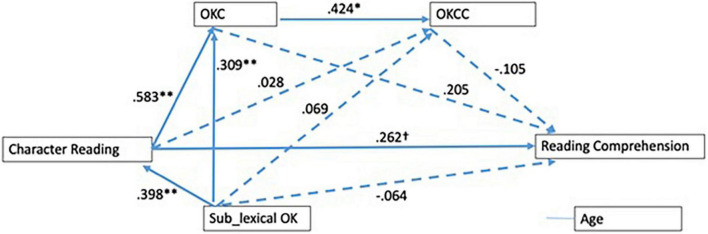
Path model demonstrating relationship between character reading and reading comprehension in the low-experienced group. **p< 0.01, *p< 0.05. ^†^Represents marginally significant (*p* = 0.053). All values were standardized coefficients. OKC, lexical orthographic choice; OKCC, lexical orthographic choice in context.

**FIGURE 3:**
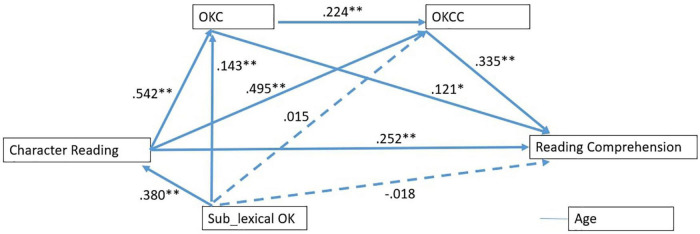
Path model demonstrating relationship between character reading and reading comprehension in in the high-experienced group. **p< 0.01, *p< 0.05. All values were standardized coefficients. OKC, lexical orthographic choice; OKCC, lexical orthographic choice in context.

A multi-group analysis was undertaken to check if mediating effects of lexical orthographic knowledge varied between two groups with different lengths of Chinese learning experience. The model shown in Figure 1 was saturated in two groups of students separately (see Figures 2,3), and then the unconstrained model (i.e., with free structural parameter coefficients) was also saturated. To conduct the multi-group analysis, we compared the constrained model (i.e., all parameters were constrained equal across groups) with the unconstrained model (Byrne, 2013). The results revealed that model fit had significantly changed [Δχ^2^(10) = 42.94, *p* < 0.001], indicating that the model fit could be varied across two groups. In the low experience group, the model accounted for a 16% variance of reading comprehension, while in the high experience group, the model explained a 37.1% variance of reading comprehension.

**TABLE 3:** The indirect effect of character reading on comprehension via two mediators.

	All sample		Low experience		High experience	
			
	Effect	95%CI	Effect	95%CI	Effect	95%CI
1.CR-OKC-RC	0.063*	[0.011, 0.121]	0.133	[−0.017, 0.342]	0.066*	[0.013, 0.124]
2.CR-OKCC-RC	0.133**	[0.072, 0.200]	−0.004	[−0.085, 0.048]	0.167**	[0.100, 0.253]
3.CR-OKC-OKCC-RC	0.036**	[0.016, 0.068]	−0.029	[−0.130, 0.022]	0.041***	[0.019, 0.081]
Total indirect effect	0.232**	[0.144, 0.324]	0.100	[−0.061, 0.300]	0.274**	[0.177, 0.384]

***p < 0.001, **p <0.01, *p < 0.05.

*CR, character reading; OKC, lexical orthographic choice; OKCC, lexical orthographic choice in context; RC, reading comprehension.*

To investigate the specific paths that varied between groups, we constructed more models by releasing the constraint one by one for each path, and compared each model with the unconstrained model using chi-square difference tests. The results indicated that all paths were invariant across groups while the other two paths had significant differences. The effect of character reading on OKCC was [Δχ^2^(1) = 10.91, *p* = 0.001], and the effect of OKCC on reading comprehension [Δχ^2^(1) = 12.11, *p* = 0.001].

Taking a closer look, we found that all indirect effects via two mediators were non-significant in the low experience group (see Table 3), but significant in the high experience group. This might suggest that as high experience CSL students developed both OKC and OKCC better, they were able to apply them to facilitate reading comprehension.

## Discussion

Along with the increasing number of CSL learners in recent decades, more attention has been devoted to exploring the role of orthographic knowledge in the development of Chinese literacy. In this study, the mediating effect of lexical orthographic knowledge in the relationship between character reading and reading comprehension was examined among a group of CSL learners in Hong Kong. The research not only enriched our understanding of literacy acquisition from character reading to reading comprehension but also provided pedagogical implications for teaching Chinese reading comprehension to second language learners.

From a bottom-up perspective, reading comprehension builds on word recognition. In response to the complex property of Chinese characters, it is believed that learners should master multiple linguistic skills necessary for successful recognition of words. Previous studies have suggested that the sub-lexical orthographic knowledge was a crucial skill in supporting learners to quickly recognize the characters among native (e.g., Ho et al., 2003) and CSL learners (Shen and Ke, 2007; Chang et al., 2014; Loh et al., 2018; Chan et al., 2021). However, because characters, as a basic unit of the Chinese writing system, only represent “morpheme” rather than “word” (Packard, 2000), it is not very clear about the exact effect of character reading on reading comprehension among CSL learners and how these learners could achieve comprehension at higher levels (i.e., word level and above).

It is tended to believe that the recognition of words relies on the analytical way of processing by integrating the morphemes (mostly characters) linearly (e.g., Ku and Anderson, 2003; McBride-Chang et al., 2003; Tong et al., 2009; Li et al., 2012; Ke and Koda, 2019), many studies were thus conducted on the related skills supporting this processing (e.g., Ku and Anderson, 2003; McBride-Chang et al., 2003; Tong et al., 2009; Li et al., 2012; Ke and Koda, 2019). While the analytical processing is surely important and indispensable, it should also be noted that due to CSL learners’ limited knowledge of Chinese morphemes, it is difficult for them to decode word meanings in an analytical way (Gong et al., 2020b). The growing number of studies have also shown that learners recognize words holistically in reading (e.g., Chu and Leung, 2005; Liu et al., 2010; Cui et al., 2021), and lexical orthographic knowledge helps to predict students’ reading comprehension performance (Leong et al., 2011). Therefore, we argued that lexical orthographic knowledge may play a vital role in CSL learners’ acquisition of reading comprehension skills.

To answer research question 1, the findings of the present study showed that lexical orthographic knowledge significantly mediated the effect of character reading on reading comprehension after controlling the effects of age and sub-lexical orthographic knowledge. As there is no salient space between Chinese words, CSL readers may develop the ability to recall their knowledge of orthographic word forms from their mental lexicon and integrate characters into words for further processing. In our study, among the three indirect effects of character reading on comprehension via two sub-types of lexical orthographic skills (i.e., OKC, OKCC), OKCC had the strongest mediation effect, indicating that CSL students’ lexical orthographic skill in sentence reading context could be a more important factor in explaining their Chinese reading comprehension performance.

Based on our results, we could also extend our discussion on the different roles played by two types of orthographic knowledge in the process of Chinese text reading among CSL learners. With reference to the process of word reading models proposed in the literature (e.g., Taft and Zhu, 1997; Li et al., 2009), readers are commonly believed to form multiple presentations at various linguistic levels during word reading, such as radical, character and word levels. In the process from radical to character level, the sub-lexical orthographic knowledge could be employed to help learners to quickly and correctly recognize the characters. Furthermore, the lexical orthographic knowledge would be subsequently involved in the process from characters to words as it helps readers to recognize words in the case that readers are already familiar with the written form of a word. However, it should be noted that the comprehension process from character level to word level is very complicated, in which character reading and word recognition could take place parallel and interactively (Li et al., 2009), therefore the sub-lexical and lexical orthographic knowledge could also function interactively with each other. Follow-up studies in this research area could be conducted, particularly among CSL learners.

In response to research question 2, we found that the mediating effects of lexical orthographic knowledge varied between different experience groups. The three paths of indirect effects were only significant in the high experience group but not the low experience group. In other words, character reading could have both direct and indirect effects on reading comprehension among high experience CSL learners, while for low experience learners, only the direct effect could be significantly observed. That might be because learners with less experience rely more on character-by-character reading (Chu and Leung, 2005) due to the insufficient orthographic word forms stored in their mental lexicon. Similar findings are also found among native Chinese learners. For example, Chen and Su (2009) and Su and Samuels (2010) pointed out that native Chinese beginning learners process words in an analytical way (i.e., reading character by character, instead of processing multi-character words as a single unit), but they are able to decode multi-character words automatically and holistically in senior grades. We assumed that CSL learners were able to gain more lexical orthographic knowledge through engaging in various learning activities (such as after-class reading), which would enable the mediating effects of lexical orthographic knowledge to function more effectively. Nevertheless, our results indicated that lexical orthographic knowledge could be an important factor in explaining the individual differences in students’ reading performance.

## Limitations and Implications

The current study has a few limitations. First, no causal associations between lexical orthographic knowledge and reading comprehension can be established from this correlational study. Meanwhile, as an initial exploration on lexical orthographic knowledge in Chinese reading comprehension, we did not take longitudinal data into account nor experimental data in the study, therefore more empirical studies should be conducted in the future. Second, the participants’ age range in the present study was wide, which may cause a confounding effect on the analysis. Third, our classification of students’ length of learning experience was based entirely on their self-reports, for which more related measurements and more robust methods (e.g., cluster analysis, latent profile analysis) could be adopted in future studies.

Despite the limitations, our study is pedagogically useful. Learning from CSL teachers, we know that learners commonly encounter difficulties in reading comprehension, though they have already mastered the knowledge of Chinese characters. We noticed that the main difficulty encountered by the students was not able to correctly identify words in a sentence. The findings in this study provide some references to address this situation. Currently, in CSL teaching, learners are commonly taught to learn single characters by rote. Previous research has suggested explicit instructions on orthographic knowledge are necessary for reading CSL (e.g., Nguyen et al., 2017; Gong et al., 2018; Ke, 2020). In light of our findings, teachers are recommended to help students better grasp the written form of words, rather than simply memorizing more characters. To be specific, teachers are suggested to adopt explicit teaching while instructing their CSL students to discern the two-character form of words. Therefore, more teaching activities related to lexical orthographic knowledge using words to form sentences are recommended.

Moreover, group differences found in our study calls for more attention to the learning experience of CSL learners. In Hong Kong, due to administrative arrangements, they are usually allocated to the same class as peers of the same age who completed their primary education in Hong Kong with a good command of conversational Chinese. To compensate the relatively less learning experience of CSL learners, more reading exposure is recommended for CSL learning. Teachers are encouraged to organize more reading activities both in the curriculum, as well as after-class activities.

## Data Availability Statement

The original contributions presented in the study are included in the article/supplementary material, further inquiries can be directed to the corresponding author/s.

## Ethics Statement

The studies involving human participants were reviewed and approved by the Education University of Hong Kong (Reference Number: EdUHK-E2019-2020-0032) and the University of Hong Kong (Reference Number: HKU-EA1502041). Written informed consent to participate in this study was provided by the participants’ legal guardian/next of kin.

## Author Contributions

EL initiated the study and designed the research instruments. XL carried out the experiments and collected the data with EL. XL and MC analyzed the data and drafted the manuscript. EL worked on the elaboration of the manuscript while maintaining close communication with XL and MC. All authors provided critical feedback, contributed to the article, and approved the submitted version.

## Conflict of Interest

The authors declare that the research was conducted in the absence of any commercial or financial relationships that could be construed as a potential conflict of interest.

## Publisher’s Note

All claims expressed in this article are solely those of the authors and do not necessarily represent those of their affiliated organizations, or those of the publisher, the editors and the reviewers. Any product that may be evaluated in this article, or claim that may be made by its manufacturer, is not guaranteed or endorsed by the publisher.

## Footnotes

1
https://www.edbchinese.hk/lexlist_ch/

## Appendix

### Chinese Character Reading

六、刃、次、文、權、師、分、火、嗎、問、下、共、漢、近、興、五、字、友、州、長、牙、龜、乙、上、田、石、女、劇、西、那、母、早、力、再、百、考、她、末、在、不、有、四、只、本、沒、白、老、毛、打、台、術、日、先、少、用、生、主、父、去、他、十、因、年、月、費、中、天、認、車、外、工、達、多、來、太、機、我、半、對、論、左、功、走、北、級、小
